# Studies of the Parasite-Midgut Interaction Reveal *Plasmodium* Proteins Important for Malaria Transmission to Mosquitoes

**DOI:** 10.3389/fcimb.2021.654216

**Published:** 2021-06-28

**Authors:** Guodong Niu, Yingjun Cui, Xiaohong Wang, Yacob Keleta, Jun Li

**Affiliations:** Department of Biological Sciences, Biomolecular Sciences Institute, Florida International University, Miami, FL, United States

**Keywords:** *Plasmodium*, malaria transmission, sexual stage, parasite-mosquito interaction, Pfs16, mosquito midgut invasion

## Abstract

Malaria transmission relies on parasite-mosquito midgut interaction. The interactive proteins are hypothesized to be ideal targets to block malaria transmission to mosquitoes. We chose 76 genes that contain signal peptide-coding regions and are upregulated and highly abundant at sexual stages. Forty-six of these candidate genes (60%) were cloned and expressed using the baculovirus expression system in insect cells. Six of them, e.g., PF3D7_0303900, PF3D7_0406200 (Pfs16), PF3D7_1204400 (Pfs37), PF3D7_1214800, PF3D7_1239400, and PF3D7_1472800 were discovered to interact with blood-fed mosquito midgut lysate. Previous works showed that among these interactive proteins, knockout the orthologs of Pfs37 or Pfs16 in *P. berghei* reduced oocysts in mosquitoes. Here we further found that anti-Pfs16 polyclonal antibody significantly inhibited *P. falciparum* transmission to *Anopheles gambiae*. Investigating these candidate proteins will improve our understanding of malaria transmission and discover new targets to break malaria transmission.

## Introduction

Malaria is a deadly infectious disease spread by mosquitoes. It affects half of the world’s population and claims nearly a half-million lives annually. Although the global malaria cases have decreased since 2010, the ten most burdened countries in Africa still had 3.5 million more cases than the previous year (Who, 2018). The fast spread of insecticide-resistant mosquitoes partially causes this. Mosquitoes become resistant to the five major chemical pesticides, making traditional vector control methods by pesticides often fail to control malaria epidemics (Alout et al., 2017). Concerns also come from the artemisinin-resistant *Plasmodium falciparum* throughout Cambodia and parts of Thailand. The spread of drug-resistant parasites may cause devastating consequences in the future (Chookajorn, 2018; Conrad and Rosenthal, 2019). Malaria vaccines that protect humans from infecting would be ideal for controlling malaria epidemics. However, no malaria vaccines have been developed. The most promising malaria vaccine candidate is RTS,S. It is mainly composed of circumsporozoite protein fragments presented on lipoprotein particles (Duffy and Gorres, 2020). However, the efficacy of RTS,S in Phase III trial was less than 36% (Rts, 2014). These issues have prompted researchers to find new strategies to stop the spread of malaria.

Since *Plasmodium*-infected mosquitoes spread malaria, a vaccine or medicine that prevents parasites from infecting mosquitoes is an alternative for malaria control. The targets of transmission-blocking reagents may participate in parasite developmental processes such as differentiation of sexual forms of microgametes and macrogametes, zygote formation, ookinete invasion of mosquito midgut, and sporozoite infection (Acquah et al., 2019). Several antigens from parasites have been examined as targets to block malaria transmission. Antibodies against Pfs25 or members of s48/45 6-Cysteine family (Pfs230, Pfs48/45, and Pfs47) on the surface of gametocytes (MacDonald et al., 2016) and zygotes (Young et al., 2005) have been shown to inhibit malaria transmission. Some proteins targeted by TBV studies were summarized in a recent review (Bennink et al., 2016).

The interaction between parasites and the mosquito midgut is a crucial determinant of successful infections in a mosquito. Quite some studies have shown mosquito proteins are essential for the ookinete formation in the mosquito midgut and their penetration of the peritrophic matrix (PM) and midgut epithelial cells. Mosquito proteins AnAPN1 and FREP1 have been identified to play roles in malaria transmission. AnAPN1 localizes on the apical surfaces of *Anopheles gambiae* midguts (Blagborough and Sinden, 2009), and studies have shown that anti-AnAPN1 antibodies significantly inhibited malaria infection in mosquitoes (Dinglasan et al., 2007; Pritsch et al., 2016). FREP1 was identified by a direct association study of clinically circulating *P. falciparum* infection in wild *An. gambiae* mosquitoes (Li et al., 2013). FREP1, located at PM, interacts with parasites, thereby facilitating malaria transmission (Zhang et al., 2015). Antibodies targeting the FREP1 FBG domain inhibited transmission of *P. falciparum* and *P. vivax* to *An. gambiae* and *An. dirus*, respectively (Niu et al., 2017). CRISPR/Cas9-mediated gene knockout of FREP1 further elucidated the function of FREP1 as a host factor in mosquitoes (Dong et al., 2018).

Parasitic proteins involved in gametogenesis, fertilization of macro- and micro-gametes, zygote-to-ookinete transformation, and penetration of the midgut endothelium are also critical for malaria transmission (Bennink et al., 2016). Recently, protein Pfs47 (PF3D7_1346800) was reported to interact with AgP47Rec in the mosquito midguts to evade mosquito immunity (Molina-Cruz et al., 2020). Also, some soluble proteins secreted by the sexual stage parasite or membrane-associated proteins such as chitinase and von Willebrand factor A-domain-related protein (WARP) are also important to complete parasite transmission to mosquitoes (Li et al., 2004). More research is required to study parasitic proteins that interact with mosquito midguts to elucidate molecular mechanisms of malaria transmission and provide more targets for malaria control.

Besides targeted by transmission-blocking vaccines, these interactive proteins can also be targeted by small molecules to block malaria transmission. The fungal secondary metabolite *P*-orlandin from *Aspergillus niger* (Niu et al., 2015), asperaculane B from *Aspergillus aculeatus* (Niu et al., 2020b), and pulixin (Niu et al., 2021) that prevent FREP1 in mosquito midgut from binding to sexual stage *Plasmodium falciparum* inhibit malaria transmission to mosquitoes. A synthetic polysulfonated polymer limits malaria transmission by inhibiting the interaction between midgut chondroitin sulfate glycosaminoglycans and ookinetes (Mathias et al., 2013).

Therefore, this study focused on sexual stage parasitic proteins that interact with mosquito midguts. These proteins should be at the cytoplasmic membrane or secreted from the parasites. The identification of these proteins will help us to understand the molecular mechanisms of malaria transmission and provide targets for vaccines and drugs to control malaria transmission.

## Materials and Methods

### Rear Mosquitoes

*An. gambiae* (G3 strain) obtained from BEI Resources was maintained in an insectary set at 28°C, 80% relative humidity, 12 hours (h) day/night cycle. Larvae were fed with grounded fish food, and adult mosquitoes were maintained with 8% sucrose solution. The commercial human (AB+, Oklahoma Blood Institute, Oklahoma City, OK) was washed with the same volume of RPMI-1640 three times by centrifugation (500xg for 5 minutes (m)). The human serum (O+, Interstate Blood Bank, Memphis, TN) was heat-inactivated at 56°C for 30 m. The red blood cells and serum were mixed at the ratio of 1:1 (v/v) to feed mosquitoes through a glass feeding device (Chemglass, Vineland, NJ) to lay eggs.

### *P. falciparum* Culture

*P. falciparum* (NF54 strain) was obtained from BEI. The parasites were cultured with the RPMI-1640 (Gibco) complete medium that contains 4% fresh O^+^ human red blood cells (Oklahoma Blood Institute, Oklahoma City, OK), 12.5 µg/mL hypoxanthine, and 10% human AB+ serum (Interstate Blood Bank, Memphis, TN) in a candle jar at 37°C. The culture was initiated at 0.25-0.5% parasitemia, and the medium was replaced daily. The day 15-17 *P. falciparum* centrifugation at 500×g for 3 m) and resuspended in human AB+ serum/packed O^+^ red blood cells (1:1 by volume) and used for standard membrane feeding assays (SMFA) (Zhang et al., 2015).

### Prediction of Transmembrane Regions in *P. falciparum* Proteins

The sequences of *P. falciparum* (3D7) proteins were downloaded from PlasmoDB. The protein sequences in fasta format were uploaded into TMHMM Server (v2.0) online (http://www.cbs.dtu.dk/services/TMHMM/). The output format parameter was “Extensive, no graphics”. The number of transmembrane regions in each protein was predicted with the TMHMM algorithm (Krogh et al., 2001).

### Selection of Candidate *P. falciparum* Proteins

The information of the signal peptide for each protein was obtained from PlasmoDB. The data of the gene expression of the published RNA-Seq data (Lopez-Barragan et al., 2011) at the different developmental stages of the parasites was downloaded from PlasmoDB. It is inside mosquito midguts, and the gametocytes become gametes that further form ookinetes. The interactions between midguts and gametocytes as well as between midguts and ookinetes are important. These genes are expected to be upregulated at the sexual stage, and their products are cytoplasmic membrane proteins or secretory proteins. Therefore, the candidate genes were selected according to the following three criteria: 1) the coding proteins containing signal peptides; 2) gene expression at sexual stages (median values of stage II gametocytes, stage V gametocytes, and ookinetes) were >5-fold higher than that at any asexual stages (maximum schizont); and 3) genes are abundantly expressed at the sexual stage with >60 RNA-Seq reads at any sexual stages (minimum value). The protein annotations, subcellular locations, and other features were obtained from the UniProt and PlasmoDB. We did not use the mass spectrometry proteomics data because it is challenging to separate cells in different stages (Swearingen and Lindner, 2018).

### Cloning and Expression of Candidate Genes With a Baculovirus Expression System in Insect Cells

Total RNA was extracted from the *P. falciparum* (NF54) infected RBCs using RNAzol (Sigma-Aldrich, MO). The cDNA was synthesized using SuperScript First-Strand Synthesis System (Invitrogen, CA). The genes were amplified by PCR using DNA Engine Dyad Thermal Cycler (Bio-Rad, CA) with gene-specific primers (**Table S1**). The DNA fragments were purified by GeneJet PCR Purification Kit (Thermo Scientific) and cloned into the modified plasmid pFastBac1, which contains a 6xHis tag at the C-terminus of the multiple cloning site. The recombinant plasmid was transformed into DH5α competent cells. PCR-positive recombinant plasmids were confirmed by sequencing and then transformed into DH10Bac competent cells to obtain recombinant bacmids. The white colonies on culture plates were picked and confirmed by PCR. About 1 μg of recombinant bacmid in 100 μL of Grace’s Insect Medium, unsupplemented (Invitrogen) was mixed with 100 μL of Grace’s Insect Medium, unsupplemented (Gibco) containing 5 μL of cellfectin II, and incubated at room temperature (RT) for 15 m. The mixture was then added to 1.5 mL of Grace’s Insect Medium, unsupplemented containing 1×10^6^ sf9 cells in a 6-well culture plate, and incubated for 4 h at 27°C. The supernatant was replaced with 2 mL of Grace’s Insect Medium (Gibco) with 10% FBS (Invitrogen) and cultured at 27°C for 3 days to produce recombinant baculoviruses. After amplification for 2-3 generations, 100 μL of the culture supernatant containing the recombinant virion was added to 2 mL of Express Five serum-free medium (Gibco) supplemented with 20 mM L-glutamine and 1 million High Five cells in the wells of a 6-well culture plate. After incubation at 27°C for three days, both supernatant and cells were harvested by centrifugation at 500×g for 5 m at 4°C. The cells were suspended in native cell lysis buffer (Invitrogen) and sonicated (pause for 30 s every 10 s) for 5 m on ice. Cell debris was removed by centrifugation (10,000 g for 5 m). The recombinant protein concentrations in culture medium and cell lysate were measured with anti-His monoclonal antibodies comparing to a standard protein concentration curve.

### Analysis of the Interaction Between a Recombinant *P. falciparum* Protein and Mosquito Midgut Lysate Using ELISA

The 3-5-day old adult female mosquitoes were fed with fresh blood using a membrane feeding device. Starting 16 h post blood meal and lasting more than 4 h, ~100 midguts were dissected from blood-engorged mosquitoes. The blood was carefully removed from the midguts. The midguts were then placed in a 1.5 mL plastic tube containing lysis buffer (50 mM Tris-HCl, 0.15 mM NaCl, 0.2% Tween-20, pH7.8) and grounded with a micro pestle (Sigma-Aldrich). Subsequently, the tissues were lysed in native cell lysis buffer (Clontech) with ultrasonication for 10 m on ice with 30 s pulse and 10 s sonication. The debris was removed by centrifugation (10,000 g for 5 m) at 4°C. The protein concentration in the supernatant was measured by the Bradford method.

About 50 µL of 1 mg/mL midgut proteins were used to coat each well on a 96 well plate at 4°C overnight. The plate was then blocked with 100 µL of 2% BSA in PBS for 2 h at RT. About 100 ng of recombinant candidate proteins in 50 µl PBS were added to each well. Each corresponding recombinant protein was heat-inactivated for 15 m at 65°C as a control. The plates were incubated for 1 h at RT. Then, 50 µl anti-His mouse monoclonal antibody (Sigma, 1:1, 000 dilutions in blocking buffer) was added and incubated at RT for 1 h, followed by incubation with 50 µl of goat anti-mouse IgG-Alkaline Phosphatase conjugate (Sigma, 1:10, 000 dilutions in blocking buffer) for 1 h at RT. Finally, 50 µL of *p*-nitrophenyl phosphate (pNPP) (Sigma-Aldrich, MO) was added to develop the plate. The plate was then washed three times with PBST (1xPBS containing 0.2% Tween 20) between two incubations. The signal was detected with an Epoch Microplate Spectrophotometer at A_405_ nm (BioTek, Winooski, VT). Because one set of data from the same sample (midgut lysate) was used multiple times (46 proteins), statistical analysis was conducted by multiple t-test using the two-stage linear step-up procedure (Benjamini et al., 2006) with 1% of False Discovery Rate (Q) implemented in GraphPad Prism 8 (GraphPad Software, USA).

### Verification of the Interaction Between a Recombinant *P. falciparum* Protein and Unhomogenized Mosquito Midguts

These six recombinant proteins were further verified by an alternative ELISA. About 10 unhomogenized midguts (collected 18 h after bloodmeal) in a 1.5 mL plastic tube were suspended in 50 µl PBS containing 100 ng of a recombinant candidate protein and incubated for 1 h at RT. The PBS buffer with chloramphenicol acetyltransferase (CAT), which was expressed with the baculovirus system in the High Five cells, was used as a blank control. Then, the midguts were collected through centrifugation (500 g for 5 m), washed with 100 µl PBS three times, and homogenized in 100 µl lysis buffer (50 mM Tris-HCl, 0.15 mM NaCl, 0.2% Tween-20, pH7.8) by a micro pestle (Sigma-Aldrich) and ultrasonication as described above. After removing the debris by centrifugation (10,000 g for 5 m) at 4°C, 50 µl of the supernatant was used to coat ELISA, followed by incubation with anti-His mouse monoclonal antibody, goat anti-mouse IgG-Alkaline Phosphatase conjugate, and pNPP as described above. The plate was washed three times with PBST (1xPBS containing 0.2% Tween 20) between two incubations. The signal was detected with an Epoch Microplate Spectrophotometer at A_405_ nm (BioTek, Winooski, VT). Statistical analysis was conducted by multiple t-test using the Two-stage linear step-up procedure (Benjamini et al., 2006) with 1% of False Discovery Rate (Q) implemented in GraphPad Prism 8 (GraphPad Software, USA).

### Antibody Transmission-Blocking Assays of *P. falciparum* Infection in *An. gambiae* Mosquitoes

*E. coli*-expression of the full-length mature Pfs16 protein and generation of the antibody was conducted by a company (Bosterbio, Pleasanton, CA). The polyclonal anti-Pfs16 antibody was generated in rabbits and purified with protein A/G immunogen affinity. The titer of the antibody was measured by coating 0.1 µg antigen to the wells in a 96-well plate and calculated by testing a series dilution of the antibody. To conduct antibody transmission-blocking assays, we suspended day 15-17 cultured *P. falciparum* with the fresh O+ type human blood to get the 0.2% final concentration of stage V gametocytes. Approximately 15 μL PBS containing 45 μg purified anti-Pfs16 rabbit polyclonal antibody was mixed with 285 μL infectious blood (final antibody concentration is 0.15 mg/ml), and an SMFA (Niu et al., 2020a) was conducted using 3-5 days old female naïve mosquitoes. After feeding for 30 m, the engorged mosquitoes were separated and maintained with 8% sugar in a BSL2 insectary (27°C, 12-h light/dark cycle, 80% humidity). Seven days after infection, midguts were dissected, stained with 0.1% mercurochrome, and examined using light microscopy to count oocysts. Equivalent amounts of irrelevant antibody or BSA were used as controls. Since the distribution of oocyst numbers does not follow the normal distribution, the results were analyzed with the nonparametric test, Wilcox-Mann-Whitney test, and the experiments were repeated three times. Also, 15 μL PBS containing 45 μg, 15 μg, 3 μg, 1 μg or 0 μg purified anti-Pfs16 rabbit polyclonal antibody were mixed with 210 μL infectious blood to conduct SMFA. Due to infection rates and mosquito mortality, >50 mosquitoes were used for infection.

### Binding Assays of Pfs16 to Mosquito Midguts

Mosquito midguts were isolated from 3-5-day-old naïve mosquitoes. The mosquito midguts were also isolated from mosquitoes 18 hr post bloodmeal. The midguts were cut in half, and the content was rinsed with 200 μL 1xPBS three times. Midguts were incubated sequentially with the recombinant Pfs16 protein (experimental group) or CAT (control group) expressed in High Five cells culture supernatant (10 μg/mL) for 1 h at RT, the rabbit antibodies against Pfs16 protein in PBS (1μg/mL) for 1 h at RT, Alexa Fluor 594-conjugated goat against rabbit antibodies (ThermoFisher, 1:500 dilutions in PBS) for 1 h in the dark at RT. The sample midguts were washed with 1XPBS 5 min for 3 times between each incubation. Finally, the treated midguts were examined under a fluorescence microscope. All midguts were exposed with the same activated light intensity, and images were taken with the same exposure time. The fluorescence pixel in images was measured with Adobe Photoshop 2020.

## Results

### Selection of *P. falciparum* Proteins That Potentially Affect Parasite Invasion in Midguts

**Figure 1:**
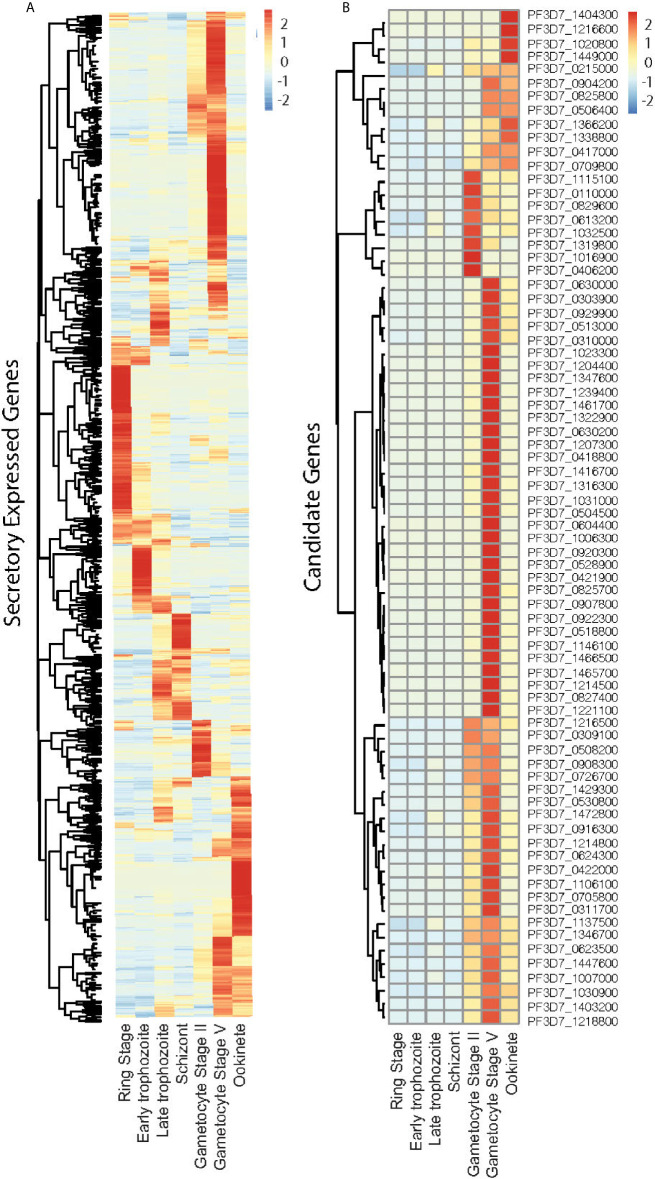
Expression profile of the *Plasmodium falciparum* genes containing signal peptide at different developmental stages. **(A)** The expression profiles of 1,079 parasitic proteins containing signal peptides using RNA-seq data (Lopez-Barragan et al., 2011). **(B)** The expression profiles of candidate genes.

We selected *P. falciparum* proteins with signal peptides, and their gene expression was upregulated and abundantly present at the sexual stage as described in methods. Based on protein sequences from PlasmoDB, 1,079 *P. falciparum* proteins contain signal peptides. The RNA-Seq data of seven developmental stages of these genes (Lopez-Barragan et al., 2011) were downloaded from PlasmoDB. Differential gene expression analysis of RNA-seq data was performed using the R package, and a heatmap was generated. The result showed stage-specific gene expression patterns, with about half of them being upregulated at the sexual stage (**Figures 1A**, **S1**). Among them, 670 gene-encoded proteins contained one or more transmembrane regions (TM), and 413 contained signal peptides without TM. We selected the genes that were >5-fold upregulated at sexual stages and abundantly expressed (>60 reads per gene) at the sexual stages (gametocyte II, V, and ookinete). These criteria were selected because only sexual stage proteins are critical for parasites to infect mosquitoes, and abundantly expressed genes have fewer measurement errors and are relatively easier to study their mechanisms. Based on the selection criteria, 76 candidate proteins were obtained for further study (**Figure 1B**). Many important genes, such as CelTOS, GEST, and SOAP, which have been proved to be necessary for parasite invasion, were included in this list (**Table 1**). The expression data from another study (Pelle et al., 2015) were used to verify the expression of candidate genes. Except for gene PF3D7_1115100, the microarray expression profiles of 75 candidate genes were obtained. Results showed that >90% of candidate genes had expression signals greater than 4, confirming their abundance at the sexual stage (**Figure S2A**). Also, the microarray data confirmed that they were upregulated at the sexual stage comparing to the asexual stage (**Figure S2B**).

**Table 1:** The features of selected candidate proteins.

*Gene ID(PF3D7)*	Expression based on RNA-seq data							*Product Description*	*TMM*	*Length (aa)*	*Location*	*C&E*
	Ring	ET	LT	ST	GII	GV	Ookinete					
*0310000*	1.14	3.09	29.91	12.51	117.95	475.94	190.3	50S ribosomal protein L9, apicoplast, putative	yes	197	apicoplast	yes
*0508200*	5.25	2.87	40.18	34.21	604.92	704.38	144.83	longevity-assurance (LAG1) protein, putative	yes	355	apicoplast	yes
*0613200*	6.5	1.67	66.31	30.45	269.35	163.88	121.91	conserved Plasmodium protein, unknown function	yes	60	apicoplast	
*0623500*	15.91	7.27	41.39	17.78	165.71	260.26	150.4	superoxide dismutase [Fe] (SOD2)	no	266	apicoplast	yes
*0726700*	4.97	6.87	41.46	6.04	325.31	367.53	121.77	conserved Plasmodium protein, unknown function	no	170	apicoplast	
*0829600*	2.32	0	14.88	2.41	401.49	193.37	98.58	early transcribed membrane protein 8 (ETRAMP8)	yes	170	apicoplast	yes
*0907800*	32.74	7.98	73.41	35.48	238.38	1960.34	307.28	ribosomal protein L35, apicoplast, putative	no	191	apicoplast	
*0908300*	6.48	2.34	83.13	39.8	424.56	486.84	150.68	conserved protein, unknown function	yes	305	apicoplast	
*0929900*	2.36	0.53	13.9	9.68	60.51	359.01	126.53	conserved Plasmodium protein, unknown function	yes	191	apicoplast	yes
*1020800*	20.15	5.42	8.46	2.58	113.51	78.63	321.75	dihydrolipoamide acyltransferase component E2	no	640	apicoplast	
*1023300*	8.5	1.91	53.69	7.74	120.29	822.3	117.75	conserved protein, unknown function	yes	79	apicoplast	yes
*1106100*	6.08	11.91	35.52	13	175.17	379.06	66.1	apicoplast ribosomal protein S15 precursor, putative	yes	269	apicoplast	yes
*1137500*	4.58	2.07	78.58	31.02	213.01	276.4	163.35	apicoplast ribosomal protein S14p/S29e precursor,	no	172	apicoplast	yes
*1472800*	4.51	1.68	71.14	23.82	145.41	355.88	130.75	HSP20-like chaperone, putative	yes	363	apicoplast	yes
*0604400*	3.8	0.86	0.7	1.15	178.99	4730.33	85.86	conserved protein, unknown function (Pfs37)	yes	178	cell surface	yes
*0904200*	5.41	1.74	1.28	0	269.16	3109.6	2284.3	PH domain-containing protein, putative	yes	292	cell surface	yes
*1204400*	3.24	1.02	1.55	0	260.54	2094.45	148.73	sexual stage-specific protein G37, putative	yes	349	cell surface	yes
*1338800*	5.14	7.23	32.17	13.49	78.81	178.26	280.73	CPW-WPC family protein	no	550	cell surface	
*0418800*	5.84	0.78	0.95	0	803.15	5476.06	624.45	MOLO1 domain-containing protein, putative	yes	261	crystalloid	yes
*0825700*	3.89	0.5	0.27	0	108.54	2480.37	580.03	crystalloid-specific PH domain-containing protein	no	305	crystalloid	yes
*1207300*	4.12	0.46	0.76	1.88	264.59	1771.22	202.82	LIMP protein, putative	no	109	crystalloid	yes
*0309100*	9.18	3.14	13.98	6.92	668.75	586.6	155.1	conserved Plasmodium protein, unknown function	no	178	cytoplasm	
*0630200*	2.08	1.13	3.07	6.07	931.97	6706.56	740.02	secreted ookinete protein, putative (PSOP6)	no	135	cytoplasm	yes
*0920300*	2.41	0.81	7.32	1.1	305.88	5919.52	334.91	conserved Plasmodium protein, unknown function	no	187	cytoplasm	yes
*0922300*	4.66	1.29	7.26	5.23	194.98	1926.68	276.13	conserved protein, unknown function	no	315	cytoplasm	yes
*1239400*	0.94	0.85	0.7	0	170.17	1061.44	107.42	conserved protein, unknown function	no	179	cytoplasm	yes
*1366200*	12.38	14.2	48.88	19.14	62.95	129.63	216.7	conserved protein, unknown function	yes	150	cytoplasm	
*1416700*	2.56	1.15	5.96	0	1072.11	4267.03	499.5	conserved protein, unknown function	yes	132	cytoplasm	yes
*1461700*	4.46	3.62	14.12	1.63	1388.9	8222.2	808.08	conserved Plasmodium protein, unknown function	no	126	cytoplasm	yes
*1465700*	2.2	0.4	0.97	0.53	63.24	1233.72	154.13	plasmepsin VIII, putative	no	385	cytoplasm	yes
*1032500*	11.58	29.21	142.95	45.35	566.79	191.19	238.5	DER1-like protein, putative (Derlin)	yes	263	endoplasmic reticulum	yes
*0422000*	6.86	8.4	20.98	1.92	116.99	318.5	70.83	steroid dehydrogenase, putative	yes	321	membrane	
*0406200*	216.87	522.9	1153.39	744.61	431324.27	52190.48	16181.79	sexual stage-specific protein precursor (Pfs16)	yes	157	membrane	yes
*0504500*	3.08	0.56	4.23	5.23	849.57	4697.28	741.87	MOLO1 domain-containing protein, putative	yes	275	membrane	
*0624300*	5.48	3.29	23.54	9.99	1668.26	3629.97	1111.5	CPW-WPC family protein	yes	185	membrane	yes
*0827400*	11.9	5.13	6.47	9.42	150.05	2537.45	477.67	conserved Plasmodium protein, unknown function	yes	218	membrane	
*0916300*	3.09	0.7	22.49	27.47	86.12	209.96	69.37	conserved Plasmodium protein, unknown function	yes	292	membrane	
*1007000*	10.63	18.78	40.9	11.86	133.55	243.47	108.27	transmembrane protein 147, putative	yes	260	membrane	yes
*1016900*	21.82	50.13	807.63	136.34	89715.85	2928.45	3224.83	early transcribed membrane protein 10.3 (ETRAMP10.3)	yes	108	membrane	
*1030900*	18.62	4.66	44.37	89.53	576.08	1043.52	694.99	ookinete surface protein P28 (Pfs28)	no	218	membrane	
*1031000*	9.35	1.64	8.8	8.52	4217.91	23349.99	3477.26	ookinete surface protein P25 (Pfs25)	yes	217	membrane	
*1403200*	3.3	0.52	1.71	0	282.14	685.68	356.55	conserved Plasmodium protein, unknown function	no	291	membrane	yes
*1216600*	4.95	1.67	3.19	6.77	90.42	64.18	1725.74	cell traversal protein for ookinetes and sporozoites (CelTOS)	no	182	microneme	yes
*1404300*	7.25	1.26	54.23	146.41	94.81	198.7	10377.13	secreted ookinete adhesive protein, putative (SOAP)	no	202	microneme	
*0518800*	4.44	0.5	0.41	0	1039.54	10083.58	1662.31	secreted ookinete protein, putative (PSOP13)	no	203	nucleus	
*0311700*	4.58	0.94	10.11	2.86	1190.93	3185.75	369.83	plasmepsin VI	yes	432	osmiophilic body	
*1214800*	5.66	0.46	5.31	1.88	1108.51	2803.96	869.86	conserved Plasmodium protein, unknown function	yes	109	osmiophilic body	yes
*1216500*	3.83	1.15	65.56	4.65	6183	5110.23	2927.77	male development gene 1 (MDV1)	no	221	osmiophilic body	yes
*1449000*	3.64	0.21	6.7	3.32	189	203.55	1188.67	gamete egress and sporozoite traversal protein (GEST)	no	248	osmiophilic body	yes
*0110000*	14.94	13.25	79.5	43.91	4159.99	1545.12	780.59	conserved Plasmodium protein, unknown function	yes	234	unknown	
*0215000*	6.07	6.28	66.04	28.19	86.63	103.68	107.44	acyl-CoA synthetase (ACS9)	no	885	unknown	
*0303900*	5.43	0	11.58	4.17	555.38	3358.77	1091.71	phosphatidylethanolamine-binding protein, putative	no	197	unknown	Yes
*0417000*	4.72	3.7	2.87	0.75	110.98	263.42	251.48	conserved Plasmodium protein, unknown function	yes	275	unknown	Yes
*0421900*	2.36	0.71	1.16	0	218.42	3456.57	358.06	conserved Plasmodium protein, unknown function	no	71	unknown	yes
*0506400*	3.39	0	3.92	1.76	113.35	1323.48	1237.32	conserved Plasmodium protein, unknown function	yes	118	unknown	yes
*0513000*	7.76	10.4	21.47	6.12	104.32	538.55	227.69	conserved protein, unknown function	no	269	unknown	
*0528900*	23.74	4.24	2.55	0.9	202.94	2764.89	251.14	conserved protein, unknown function	no	228	unknown	
*0530800*	12.22	6.61	57.56	33.19	1237.56	2048.45	289.69	CPW-WPC family protein	no	254	unknown	yes
*0630000*	4.03	0.55	0.74	0	157.87	1484	442.85	CPW-WPC family protein	yes	289	unknown	yes
*0705800*	4.38	2.11	4.08	0	682.37	1631.31	286.22	cysteine-rich secretory protein, putative	yes	193	unknown	yes
*0709800*	13.07	4.71	14.76	3.18	61.39	89.27	106.78	conserved protein, unknown function	yes	64	unknown	
*0825800*	5.58	1.03	0.93	0	105.54	2523.89	2355.77	conserved protein, unknown function	no	446	unknown	
*1006300*	7.47	2.13	1.45	0	301.55	4881.44	354.62	conserved Plasmodium protein, unknown function	no	143	unknown	
*1115100*	23.35	8.62	26.54	34.18	1085.19	398.79	415.29	conserved protein, unknown function	no	307	unknown	yes
*1146100*	3.86	0	4.26	0	995.55	7107.6	1081.7	conserved protein, unknown function	no	175	unknown	
*1214500*	9.99	9.56	4.46	1.1	258.57	5263.68	705.22	conserved Plasmodium protein, unknown function	yes	186	unknown	yes
*1218800*	3.72	2.04	7.27	1.18	108.64	259.86	120.4	secreted ookinete protein, putative (PSOP17)	no	349	unknown	
*1221100*	1.74	0.98	6.23	2.37	150.63	3971.38	628.74	conserved Plasmodium protein, unknown function	no	260	unknown	
*1316300*	7.01	0.97	0.79	0	675.09	3205.13	533.55	conserved Plasmodium protein, unknown function	no	104	unknown	yes
*1319800*	5.04	1.05	7.14	1.41	5387.24	2344.32	112.06	conserved Plasmodium protein, unknown function	yes	145	unknown	yes
*1322900*	49.77	16.63	27.17	14.07	336.07	2473.53	292.96	conserved protein, unknown function	yes	263	unknown	
*1346700*	3.91	0.45	3.9	0.92	109.52	128.45	80.61	6-cysteine protein (P48/45)	yes	448	unknown	
*1347600*	4.48	0.92	10.95	4.45	181.53	1539.74	108.79	conserved protein, unknown function	yes	277	unknown	yes
*1429300*	5.94	7.41	37.22	6.1	1131	1842.23	378.5	CPW-WPC family protein	no	371	unknown	yes
*1447600*	5.57	1.26	2.73	0	373.24	687.62	312.91	conserved protein, unknown function	yes	121	unknown	yes
*1466500*	6.74	0	1.99	0	501.1	3660.75	692.51	conserved Plasmodium protein, unknown function	no	126	unknown	yes

ET, early trophozoite; LT, late trophozoite; ST, Schizont; GII, stage II gametocyte; GV, stage V gametocyte; C&E, successfully cloned and expressed.

Because not all membrane proteins have signal peptides, we also investigated the parasitic proteins that had TM while lacking signal peptides. About 949 *P. falciparum* proteins have TM and lack signal peptides (**Table S2**). However, none of them satisfied the expression criteria, e.g., >5-fold upregulated at sexual stages and abundantly expressed at the sexual stages (>60 reads per gene).

**Figure 2:**
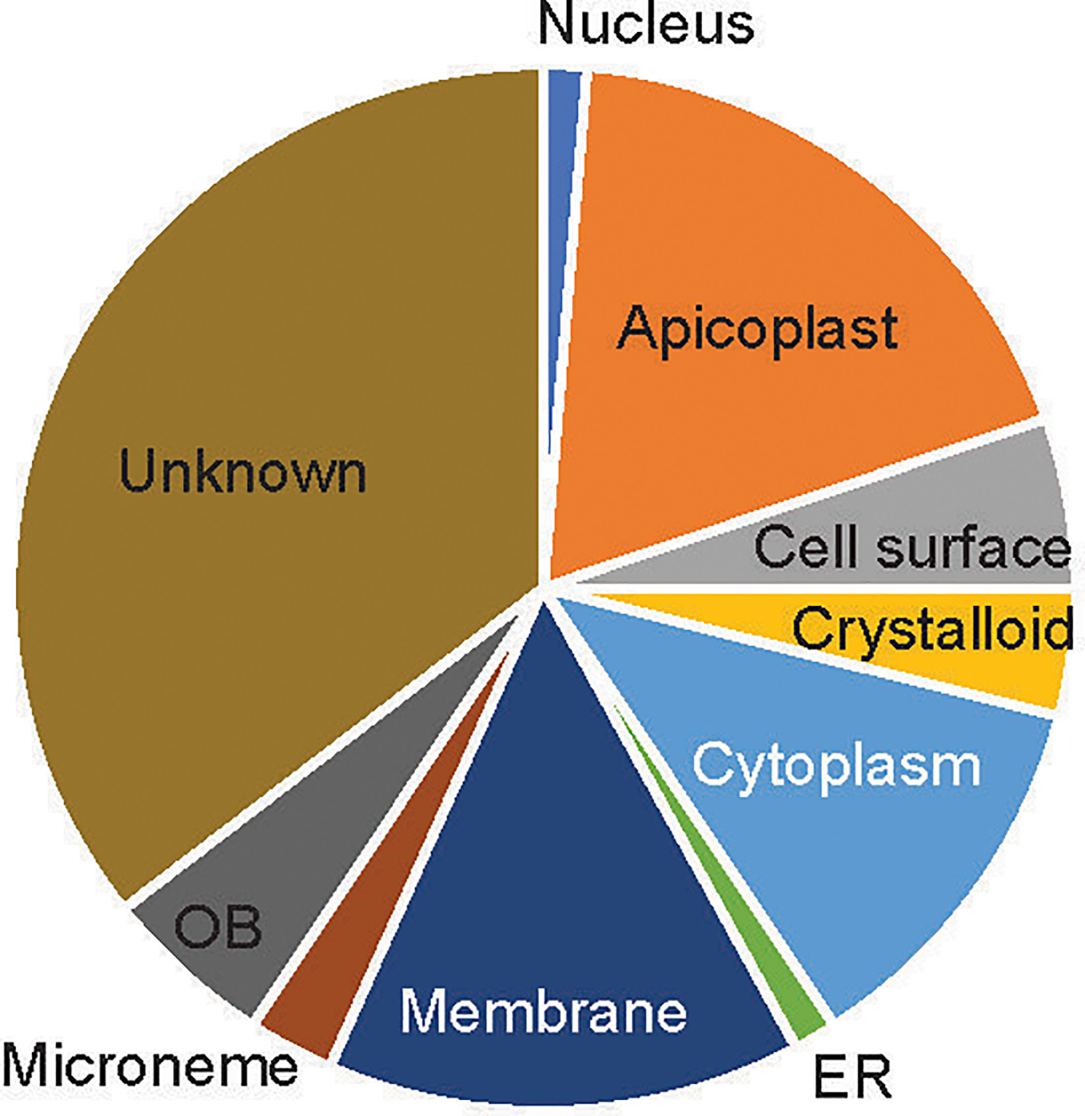
The subcellular location of the candidate proteins. ER, endoplasmic reticulum; OB, osmiophilic body.

About 39 of the 76 candidate proteins contained TM. These 76 candidate proteins were localized in different subcellular compartments, although subcellular locations of 35% of these proteins (n=27) are still unknown (**Figure 2**). The rest were distributed in 9 different locations, including rhoptry neck, apicoplast, cell surface, crystalloid, cytoplasm, endoplasmic membrane, microneme, nucleus, and osmiophilic body. Among them, 18% of these proteins (n=14) are located on the apicoplast. The apicoplast is a specific organelle important for *Plasmodium* development. Also, a decent number of candidate proteins are located on the membrane (15%), which include parasitophorous vacuole membrane and cytomembrane.

### Determination of Candidate *P. falciparum* Proteins That Interact With the Mosquito Midgut

**Figure 3:**
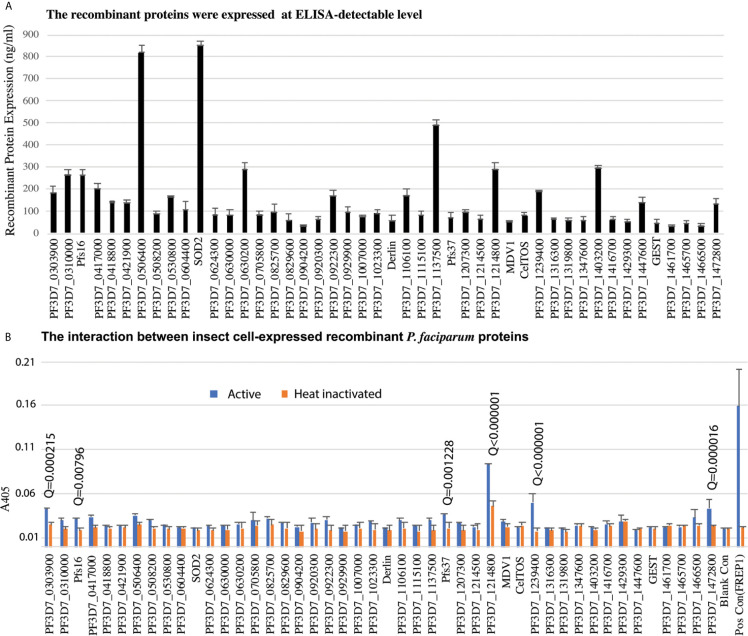
The insect-cell expressed recombinant parasitic proteins and their interaction with midguts. **(A)** The expression of candidate proteins in high five cells. Totally, forty-six genes were expressed in high five cells with a baculovirus expression system at a detectable level. Each sample was measured with triplicates. The expression experiments were conducted three times. **(B)** Interaction between recombinant proteins and midgut lysate. The concentration of recombinant proteins was normalized. Naïve recombinant proteins and heat-inactivated proteins were used as experimental groups and control groups with triplets. The false discovery rates (Q) were calculated by multiple t-test using the Two-stage linear step-up procedure (Benjamini et al., 2006). Those proteins with Q <1% were labeled with the Q values. The same experiments were repeated three times, and the results were similar.

Candidate genes were PCR-cloned and the recombinant proteins were expressed in High Five insect cells using the baculovirus expression system in the serum-free medium. A monoclonal antibody against the 6xHis tag at the C-terminus of recombinant proteins was used to quantify the recombinant protein concentration. We have finally cloned and expressed 46 genes successfully at ELISA detectable level for further analysis (**Figure 3A**).

To determine if *P. falciparum* candidate proteins interacted with *An. gambiae* midgut lysate, we performed ELISA. Mosquitoes were fed with blood, and their midguts, including peritrophic matrix, were isolated 15-21 h post blood meal. A 96-well 100 ng of recombinant protein was added into wells to determine the interaction between a candidate protein and the midgut lysate. The heat-inactivated proteins were used as the corresponding negative controls. Both the experimental group and control were done in triplicates per treatment. CAT, which was expressed with the baculovirus system in the High Five cells, was used as a blank control. The A_405_ of experimental and control wells were measured (**Figure 3B**). Since the same samples were used in 46 genes, the A_405_ values were analyzed with multiple t-test using the two-stage linear step-up procedure (Benjamini et al., 2006) with 1% of False Discovery Rate (Q). The result showed that six *P. falciparum* proteins (PF3D7_0303900, PF3D7_0406200 (Pfs16), PF3D7_1204400 (Pfs37), PF3D7_1214800, PF3D7_1239400, PF3D7_1472800) were retained by midgut lysates at a level significantly higher than the corresponding controls (**Figure 3B**, False Discovery Rate (Q) < 0.01).

**Figure 4:**
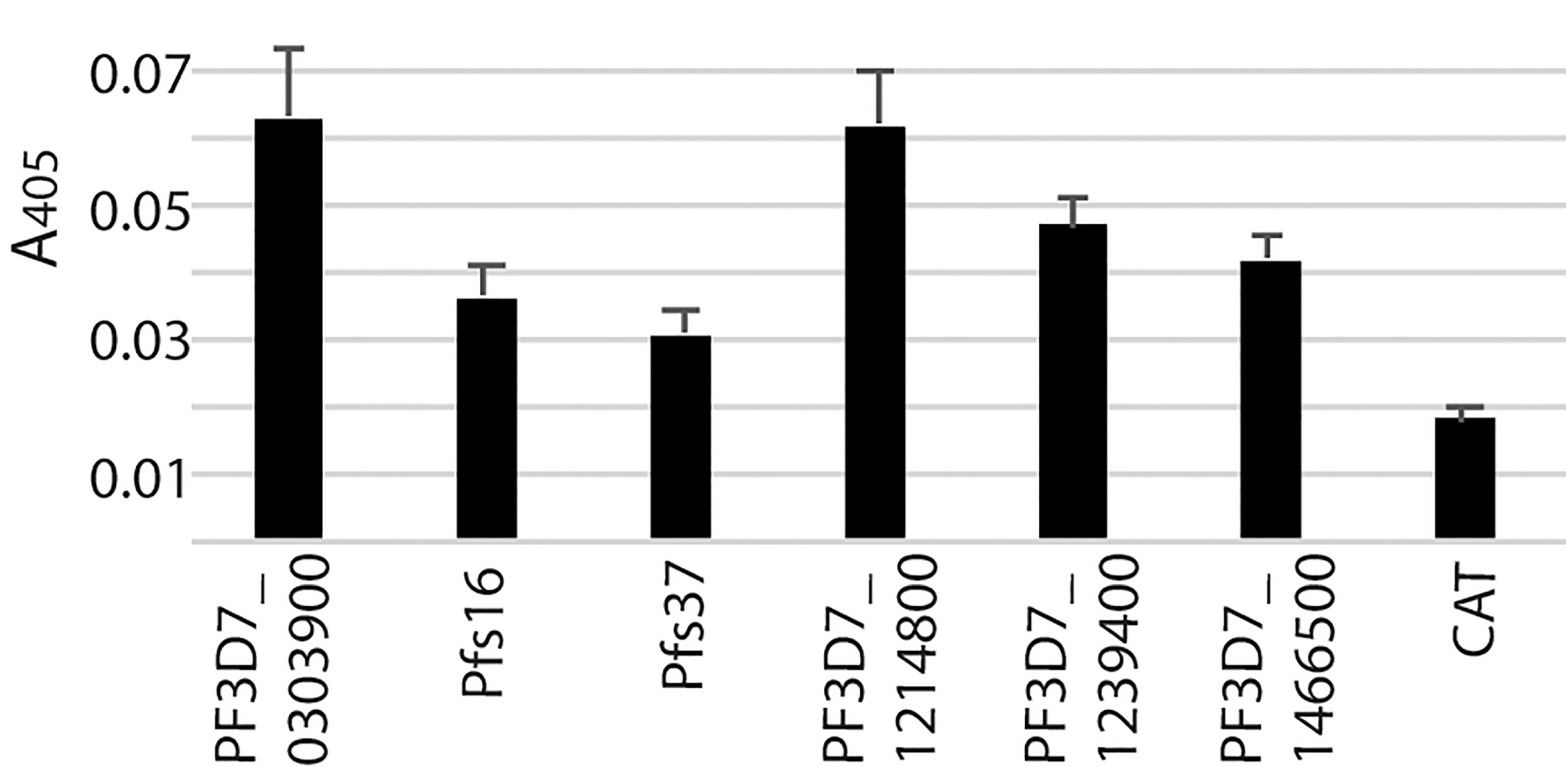
The interaction between the candidate proteins and unhomogenized mosquito midguts.

These six recombinant proteins were further verified by an alternative ELISA using unhomogenized midguts to pull down the recombinant proteins following by homogenizing and detection. The results (**Figure 4**) showed the difference between each recombinant protein and the blank control was significant (Q < 0.01), which confirmed the interaction between the candidate proteins and midguts.

### Functional Analysis of *P. falciparum* Proteins That Bound to Mosquito Midguts

To understand the function of candidate proteins, we searched their orthologs in *P. berghei* and analyzed the *P. berghei* infectious phenotypes for their knockout in public databases RMgmDB (Khan et al., 2013; Gomes et al., 2015), PlasmoGEM (Bushell et al., 2017), and other works (Zhang et al., 2018). Results (**Table 2**) showed that three *P. berghei* genes were knocked out successfully, and two *P. falciparum* genes could be mutated by transposon insertions (Zhang et al., 2018). Disruption of orthologs of PF3D7_12148, PF3D7_1204400 (Pfs37) or PF3D7_0406200 (Pfs16) did not change *P. berghei* infection at asexual stage. Knockout of PBANKA_1430600, the ortholog of PF3D7_1214800, did not affect asexual stage parasites, and the effect on sexual stage parasites and transmission has not been reported yet. Knocking out the orthologs of Pfs37 or Pfs16 rendered *Plasmodium* parasites to generate fewer oocysts in mosquitoes. Pbg37, the ortholog of Pfs37, is a protein of 37 kDa with a signal peptide and multiple transmembrane domains and only expressed at sexual stages of parasites such as gametocytes, zygotes, and ookinetes. Knockout of Pbg37 led to reduced gametocyte production and significantly affected the further development of ookinetes from gametocytes. Notably, the anti-Pbg37 antiserum from the immunized mice significantly reduced the number of oocysts in the midgut, which indicates that it could be a target against malaria infection (Liu et al., 2018).

**Table 2:** P*. berghei* phenotypes of candidate gene knockouts.

Gene ID(*PF3D7*)	Pb ID(*PBANKA*)	Coverage%	Identity%	The function of gene products	# of TM	location	knock out	Phenotype				
								AS	GAM	OK	OC	SP
*1239400*	*1453900*	96	45	unknown	0	cytoplasm	fail/mutable					
*1214800*	*1430600*	97	35	unknown	1	osmiophilic body	succeed	ND				
*1204400*	*0603300*	97	64	sexual stage-specific protein G37 (Pfs37)	7	cell surface	succeed	ND	ATTN	ATTN	ATTN	
*0406200*	*1003900*	35	26	sexual stage-specific protein (Pfs16)	2	membrane	succeed	ND	ND	ND	ATTN	ND
*0303900*	*0402500*	96	53	phosphatidylethanolamine-binding protein	0	unknown	fail/mutable					
*1472800*	*1336000*	72	60	HSP20-like chaperone	1	cytoplasm	fail/nonmutable					

AS, asexual stage; GAM, gametocyte; OK, ookinete; OC, oocyst; SP, sporozoite.

ND, no difference; ATTN, the number of the knocked parasite was attenuated compared with wild type strain. “Fail/succeed” was based on individual gene knockout. Mutable/nonmutable was based on transposon mutagenesis (Zhang et al., 2018).

Next, we selected Pfs16 to study the antibody effects on malaria transmission. Pfs16 was found in the *P. falciparum* gametocyte parasitophorous vacuole membrane. Knockout of PBANKA_1003900 (Pbg16), the ortholog of Pfs16 in *P. berghei*, did not change *Plasmodium* asexual stage (Kongkasuriyachai et al., 2004) or formation of ookinetes (Deligianni et al., 2018). Disruption of Pbg16 reduced the number of oocysts in mosquitoes (**Table 2**) (Kongkasuriyachai et al., 2004). However, the amino acid sequences of Pfs16 and Pbg16 are largely different. Thus, we further examined the function of Pfs16 on parasite transmission using transmission-blocking assays.

**Figure 5:**
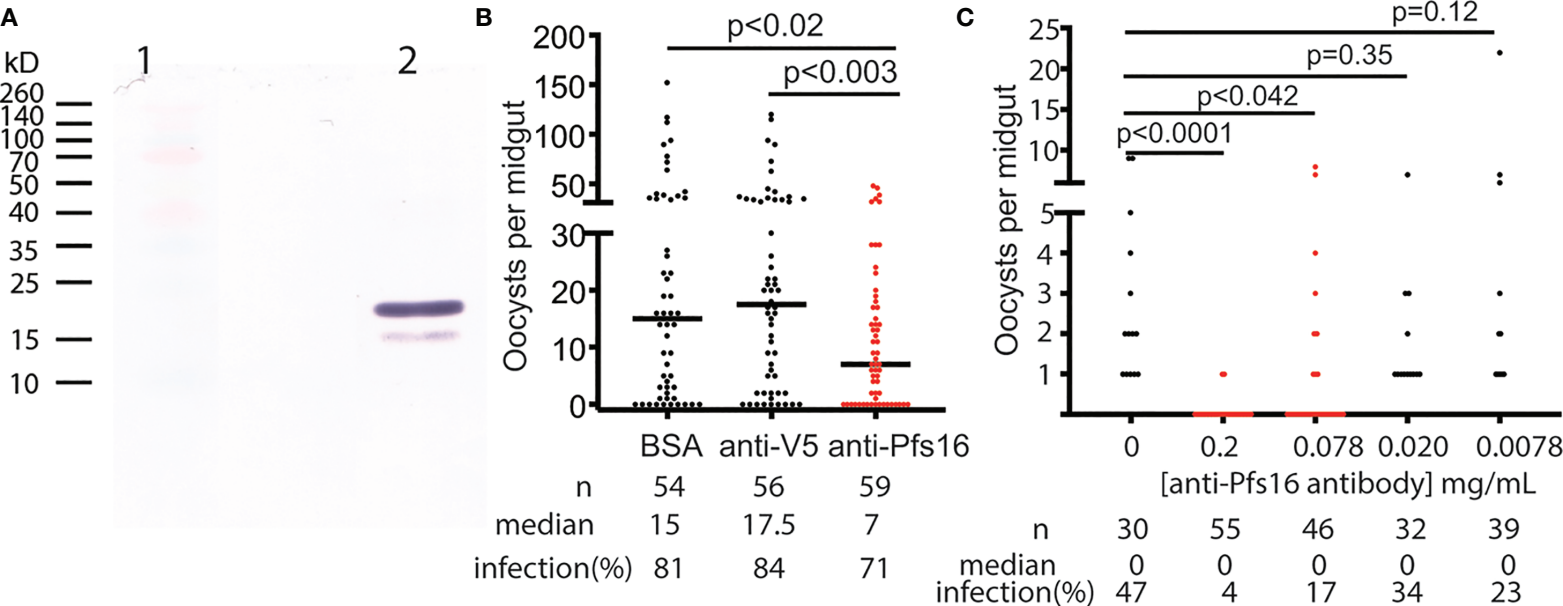
The functional analysis of Pfs16 on *Plasmodium* transmission to mosquitoes. **(A)** Western blot assay showed that anti-Pfs16 antibody specifically recognized the insect cell-expressed recombinant Pfs16 protein in lane 2. Two bands in lane 2 are the precursor and mature forms of Pfs16 with His- and V5-tag Lane 1 displayed standard protein markers with some color proteins. **(B)** Rabbit anti-Pfs16 polyclonal antibody inhibited *P. falciparum* transmission to *An. gambiae* by SMFA. The same concentration of BSA and an unrelated antibody (anti-V5) were used as the controls. The p-values between the experimental and the control groups were calculated using Wilcoxon-Mann-Whitney tests. The assays were repeated, and the results were similar. Horizontal bars are the median numbers of oocysts per mosquito midguts. **(C)** The antibody inhibition on malaria transmission was dose-dependent. This experiment was repeated twice, and the results were similar.

The anti-Pfs16 polyclonal antibody was generated in rabbits immunized with *E. coli*-expressed Pfs16 as the antigen and further purified using protein A/G immune affinity column. The specificity of the anti-Pfs16 antibody was verified with Western blot analysis using the recombinant protein expressed in insect cells. The result (**Figure 5A**) showed two bands with the size of about 19kD and 16kD, which matched the precursor (with signal peptide) and mature forms of recombinant protein containing 6xHis and V5 tags. Therefore, a rabbit polyclonal antibody against Pfs16 could specifically recognize recombinant Pfs16. Next, we determined the inhibition of anti-Pfs16 polyclonal antibody against *P. falciparum* transmission to mosquitoes. The *P. falciparum* culture containing 0.2% stage V gametocytes and 0.15 mg/ml anti-Pfs16 antibody (titer = 10^3^/mg) was used to infect *An. gambiae* (G3) using SMFA. The equal amounts of unrelated polyclonal antibody (anti-V5) or BSA were used to substitute anti-Pfs16 antibody as the control. The results showed that compared with the anti-V5 or BSA controls, the anti-Pfs16 Ab significantly reduced the number of oocysts by 50% and 57%, respectively (**Figure 5B**), supporting that Pfs16 is a potential target for malaria control. This experiment was repeated three times and obtained similar results. We also examined the different concentrations (0.2 mg/mL, 77 μg/mL, 20 μg/mL, 7.7 μg/mL and 0 μg/mL) of anti-Pfs16 on *P. falciparum* transmission to *An. gambiae* using SMFA. The results showed that the antibody inhibition rate decreased with the reduction of antibody concentration (**Figure 5C**). When antibody concentration was greater than 0.078 mg/mL, the inhibition against malaria transmission to mosquito was significant (p < 0.05).

### Recombinant Pfs16 Protein Bound to Blood-Fed Mosquito Midguts

**Figure 6:**
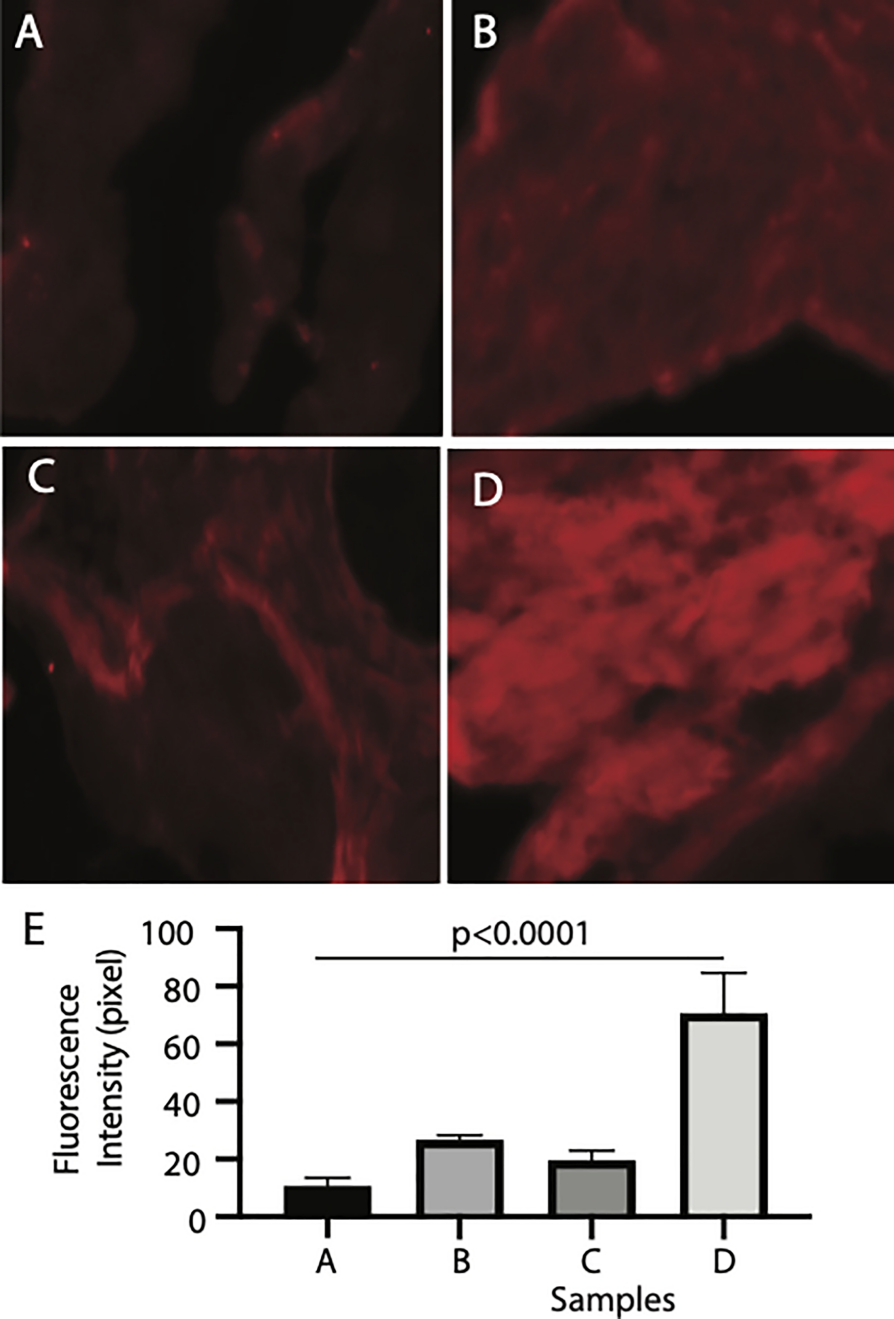
Recombinant Pfs16 protein bound to blood-fed mosquito midguts. **(A)** The naïve mosquito midguts were incubated with unrelated protein (CAT). **(B)** The blood-fed mosquito midguts incubated with un-related protein (CAT). **(C)** The naïve mosquito midguts incubated with Pfs16. **(D)** The blood-fed mosquito midguts incubated with Pfs16 protein. **(E)** The median fluorescence intensity of differently treated midguts. P. Calculated with one-way ANOVA.

Finally, we examined the binding tissues of Pfs16. The naïve mosquito midgut contains endothelia and the basal lamina, while a peritrophic matrix was formed after a blood meal. The mosquito midguts were isolated from the naïve and blood-fed mosquitoes and incubated with insect cell-expressed recombinant Pfs16 protein or the non-specific insect cell-expressed protein (CAT) as the control. The rabbit polyclonal antibody against Pfs16 was used to detect the bound Pfs16. Results showed that the fluorescence from naïve mosquitoes incubated with CAT was the weakest (**Figure 6A**). Substitution of the CAT with Pfs16 protein resulted in a slighter increase of fluorescence intensity (**Figure 6C**); however, it was similar to the blood-fed mosquito midguts incubated with CAT and anti-Pfs16 (**Figure 6B**). The fluorescence was the highest for the blood-fed mosquito midguts incubation with Pfs16 and anti-Pfs16 (**Figure 6D**). The medians of fluorescence intensity of four treatments were shown in **Figure 6E**. Collective, these data suggest that Pfs16 protein interacted with the midgut peritrophic matrix.

## Discussion

Mosquito midgut is the first place for *Plasmodium* development into ookinetes and the subsequent invasion. The interaction between *Plasmodium* parasites and mosquito midgut plays a key role in malaria transmission (Zhang et al., 2015). However, the parasite-midgut interactome is not well-understood. This project investigated parasitic secreted proteins that interact with mosquito midguts.

About 76 parasitic proteins with signal peptides and abundant at the sexual stage of parasites were selected for investigation. Many of these candidate proteins reportedly play essential roles in malaria transmission. For instance, the CelTOS is essential for ookinetes to rupture the midgut epithelial cell membrane (Kariu et al., 2006), and GEST is the gamete egress and sporozoite traversal protein vital for gametes exit hosts in mosquito midguts (Stone et al., 2018). Our data showed that both CelTOS and GEST did not directly bind to the mosquito midguts, consistent with the reported mechanisms that CelTOS was secreted directly into endothelial cytoplasm (Kariu et al., 2006) and PEST ruptured gametocyte parasitophorous vacuole membrane (Talman et al., 2011).

The 76 candidate proteins locate at different subcellular locations, such as apicoplast, osmiophilic body, and microneme. Previous studies have shown that the abundant proteins in apicoplast have been often the targets to develop malaria drugs (Biddau and Sheiner, 2019). Micronemes play a critical role during the invasion of parasites in mosquitoes (Li et al., 2004). Three ookinete-secreted proteins, PgCHT1 chitinase, WARP, and CTRP from micronemes, can break mosquito midgut to facilitate malaria transmission. Antibodies against these proteins interfere with parasite invasion (Li et al., 2004). There are several membrane proteins e.g., Pfs25 (PF3D7_1031000), P28 (PF3D7_1030900), and P48/45 (PF3D7_1346700) that under investigation as TBV by communities.

In the meantime, our bioinformatics analysis might miss some genes if they lack signal peptides or their expression data were incomplete. For instance, mosquito AgP47Rec was recently reported to interact with parasitic protein PF3D7_0134800 (P47) (Molina-Cruz et al., 2020). It was missed in our candidates because the RNA-seq expression data lacks its expression at schizonts (Lopez-Barragan et al., 2011).

Out of the 76 candidate genes, 30 were not cloned into the plasmid due to incorrect annotation or were cloned into plasmids with low expression in insect cells. This is because some proteins such as SOAP rapturing insect cells and some membrane proteins are hard to clone and express in insect cells. Notably, forty-six genes (60.5%) were successfully cloned and highly expressed with a baculovirus expression system in High Five cells. By screening these proteins *via* ELISA analysis, we identified six *P. falciparum* proteins that interacted with the midgut of mosquitoes. The orthologs of these six *P. falciparum* genes were found in *P. berghei*. Knockout of Pbg37 and Pbg16, orthologs of Pfs37 and Pfs16, respectively, from *P. berghei* reduced *Plasmodium* oocysts in mosquitoes (Kongkasuriyachai et al., 2004; Liu et al., 2018). Disruption of the Pbg37 gene negatively impacted malaria transmission, which might be caused by attenuated gametocytes. Importantly, the anti-Pbg37 antiserum inhibited *P. berghei* transmission to mosquitoes (Liu et al., 2018), supporting Pfs37 is an excellent target to break malaria transmission.

Knockout of the Pbg16 gene did not change parasites at the sexual stage, and the formation of ookinetes was normal. The amino acid sequences between Pfs16 and Pbg16 are strikingly different, with only 35% Pfs16 was covered by Pbg16, and covered regions shared 26% identical amino acids. Therefore, we generated polyclonal antibodies to examine Pfs16 function in malaria transmission. Our data show that the ingested antibodies against Pfs16 significantly inhibited parasite transmission to mosquitoes. This result supports that Pfs16 is accessible to extracellular proteins such as antibodies. Pfs16 was observed as a gene at the onset of *P. falciparum* gametocytogenesis and often used as a marker for the production of the sexual stage of parasites (Bruce et al., 1994; Dechering et al., 1997; Lanfrancotti et al., 2007). Pfs16 seems not essential in the development of gametocytes and is not involved in the formation of zygote and ookinetes. However, it may play some role in the optimal production of sexual parasites. During the entire gametocyte maturation from stages I–V Pfs16 (Lobo et al., 1994; Dechering et al., 1997; Berry et al., 2009), it was detected to be localized on the parasitophorous vacuole membrane (Moelans et al., 1991; Baker et al., 1994; Eksi and Williamson, 2011). One publication reported that anti-Pfs16 antisera did not show any transmission-blocking activities. However, no data were presented in that paper (Moelans et al., 1995). Differently, our results showed that the purified anti-Pfs16 polyclonal antibody (0.15 mg/mL) significantly inhibited parasite transmission to mosquitoes.

Successful parasite invasion of mosquitoes begins with parasites’ interaction with mosquito midguts. Through bioinformatics analysis followed by protein interaction assays, we identified six midgut-binding parasitic proteins. Moreover, we showed that antibodies against Pfs16 inhibited malaria transmission. Further investigation of these proteins will improve our understanding of the molecular mechanisms of malaria transmission and provide targets to break malaria transmission.

## Data Availability Statement

The original contributions presented in the study are included in the article/**Supplementary Material**. Further inquiries can be directed to the corresponding author.

## Author Contributions

GN and YC conducted experiments, interpreted the data, and wrote the manuscript draft. XW conducted bioinformatics and statistical analysis. YK expressed Pfs16 proteins in insect cells. JL conceived concepts, designed the project, and wrote the manuscript. All authors contributed to the article and approved the submitted version.

## Funding

This work is supported by NIAID (No. 1R01AI125657) and NSF Career Award (No. 1453287). The funders had no role in study design, data collection and analysis, decision to publish, or preparation of the manuscript.

## Conflict of Interest

The authors declare that the research was conducted in the absence of any commercial or financial relationships that could be construed as a potential conflict of interest.

## Supplementary Material

Supplementary Table 1 Specific primers for candidate genes.

Supplementary Table 2 The expression data of genes that contain transmembrane domains and lack signal peptides.

Supplementary Figure 1 The heatmap of 1,079 parasitic proteins containing signal peptides.

Supplementary Figure 2 The expression of parasitic proteins based on microarray showing the abundance of candidate genes at sexual stages.
