# Supplementary figures and images for: Studies of the Parasite-Midgut Interaction Reveal Plasmodium Proteins Important for Malaria Transmission to Mosquitoes
Source: Front Cell Infect Microbiol. 2021 Jun 28;11:654216. doi: 10.3389/fcimb.2021.654216 (PMC8274421; doi:10.3389/fcimb.2021.654216)

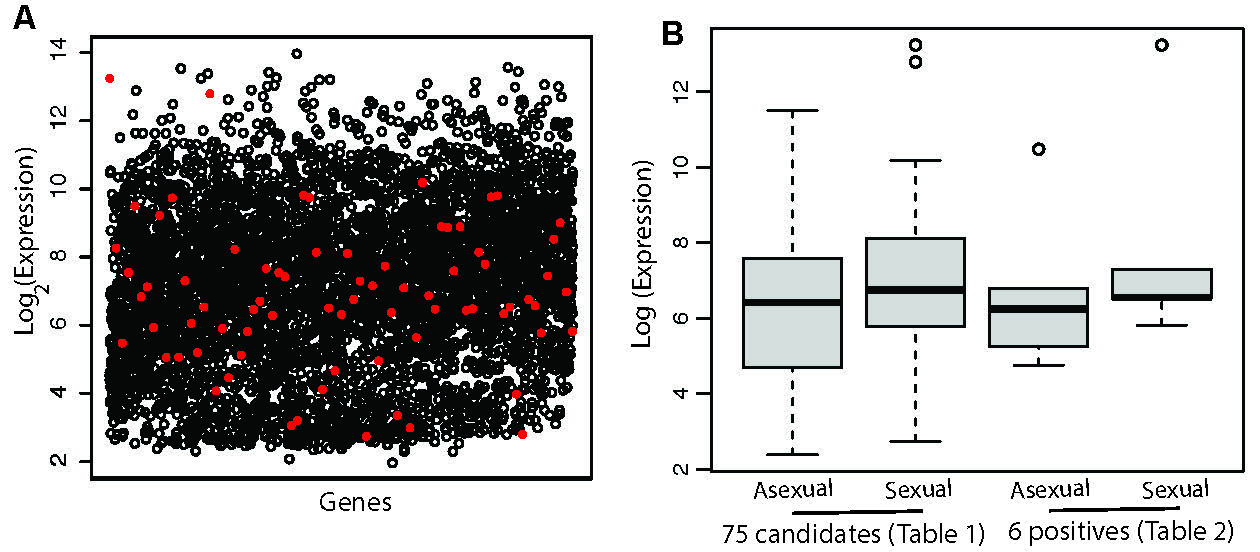

Supplement: Supplementary Figure 1 — The heatmap of 1,079 parasitic proteins containing signal peptides. [file Image_1.jpeg]

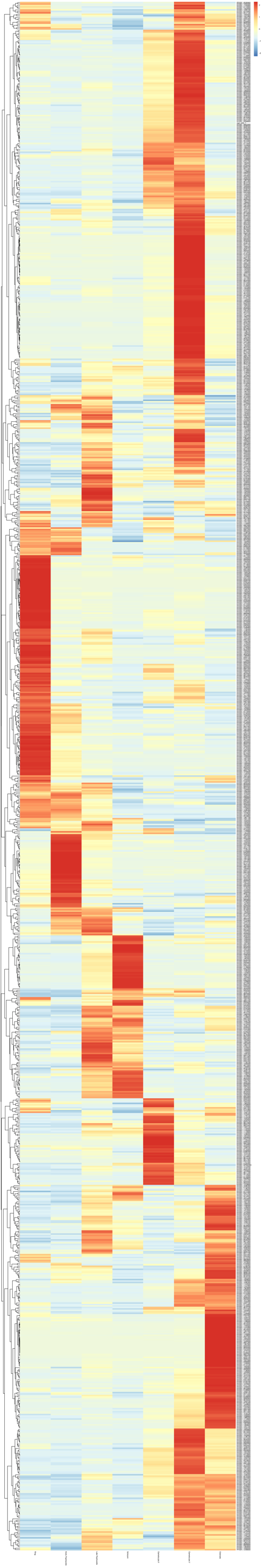

Supplement: Supplementary Figure 2 — The expression of parasitic proteins based on microarray showing the abundance of candidate genes at sexual stages. [file DataSheet_2.pdf]
